# Modulation of Gut Microbiota and Immune System by Probiotics, Pre-biotics, and Post-biotics

**DOI:** 10.3389/fnut.2021.634897

**Published:** 2022-01-03

**Authors:** Yue Liu, Jiaqi Wang, Changxin Wu

**Affiliations:** ^1^Key Lab of Medical Molecular Cell Biology of Shanxi Province, Institutes of Biomedical Sciences, Shanxi University, Taiyuan, China; ^2^The Provincial Key Laboratories for Prevention and Treatment of Major Infectious Diseases Shanxi, Institutes of Biomedical Sciences, Shanxi University, Taiyuan, China

**Keywords:** probiotics, pre-biotics, post-biotics, gut microbiota, immune system

## Abstract

The human gastrointestinal tract harbours a complex microbial community, which interacts with the mucosal immune system closely. Gut microbiota plays a significant role in maintaining host health, which could supply various nutrients, regulate energy balance, modulate the immune response, and defence against pathogens. Therefore, maintaining a favourable equilibrium of gut microbiota through modulating bacteria composition, diversity, and their activity is beneficial to host health. Several studies have shown that probiotics and pre-biotics could directly and indirectly regulate microbiota and immune response. In addition, post-biotics, such as the bioactive metabolites, produced by gut microbiota, and/or cell-wall components released by probiotics, also have been shown to inhibit pathogen growth, maintain microbiota balance, and regulate an immune response. This review summarises the studies concerning the impact of probiotics, pre-biotics, and post-biotics on gut microbiota and immune systems and also describes the underlying mechanisms of beneficial effects of these substances. Finally, the future and challenges of probiotics, pre-biotics, and post-biotics are proposed.

## Introduction

**Figure 1:**
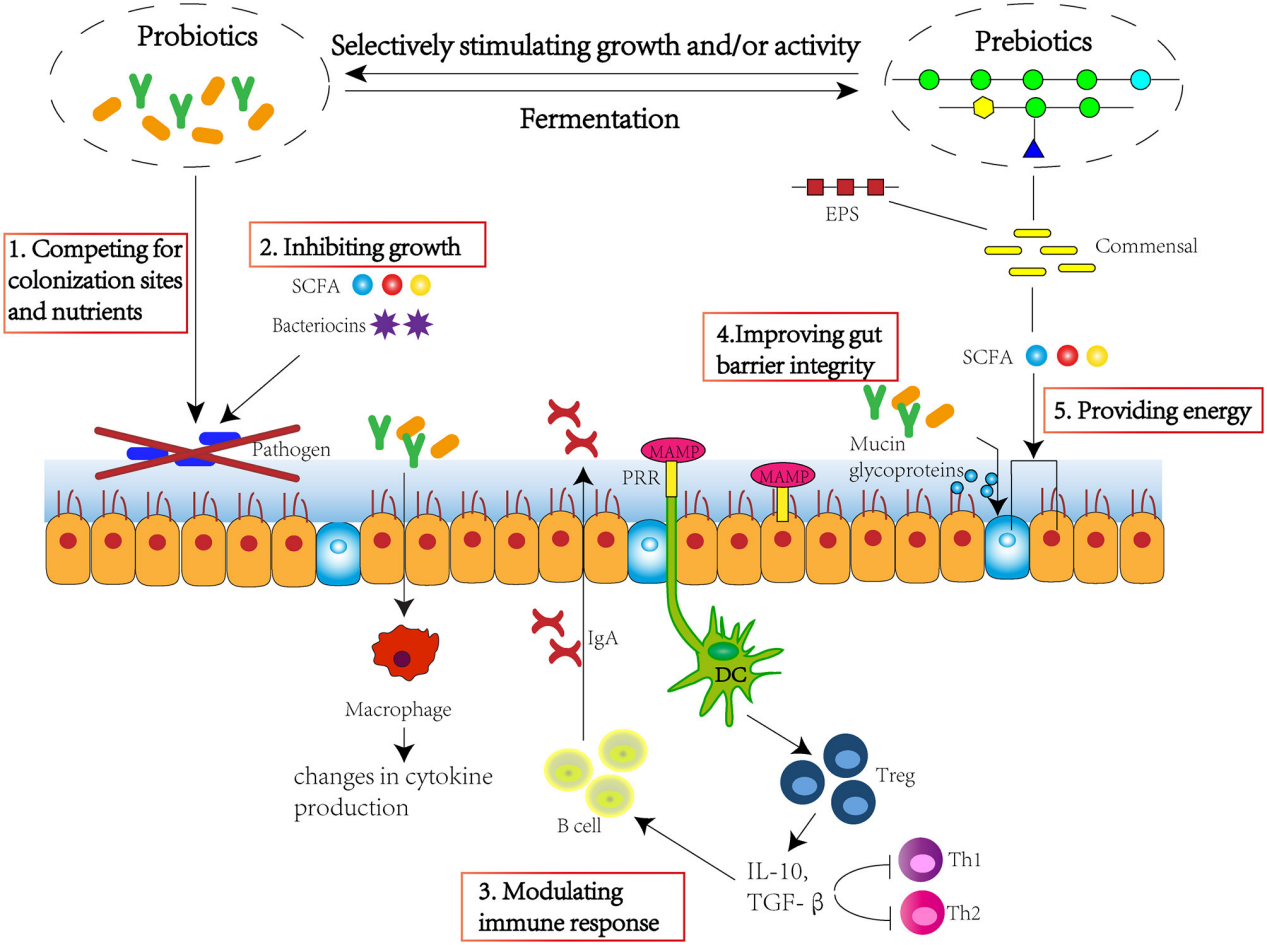
Pre-biotics, probiotics, and post-biotics are able to improve intestinal microbiota homeostasis, maintain gut barrier integrity, and modulate immune response. The approach mechanisms include 1. competing for colonisation sites and nutrients; 2. inhibiting growth through production of SCFA and bacteriocins; 3. modulating immune response through stimulating immune cells and cytokine and immunoglobulin production; 4. improving gut barrier integrity through stimulating the production of mucin glycoproteins; 5. providing energy for epithelium, improving villi growth, crypt development, tight junctions, and mucin production. SCFA, short-chain fatty acid; EPS, exopolysaccharide; MAMP, microorganism-associated molecular patterns; PRR, pattern recognition receptor; DC, dendritic cell; Treg, regulatory T cell; IL-10, interleukin 10; TGF-β, transforming growth factor β.

Over 1,000 bacterial species exist within the human gut, with more than 50 bacterial genera being described (1). The large intestine harbours the highest numbers of bacteria in the gastrointestinal tract, at around 10^11^-10^12^ cells per gramme (2). Gut microbiota plays a significant role in maintaining host health, which could supply various nutrients, regulate energy balance, modulate the immune response, and defence against pathogens (3). Therefore, maintaining a favourable equilibrium of gut microbiota is beneficial to host health. Several studies have found that pre-biotics, probiotics, and probiotic-driven metabolites, known as “post-biotic,” are able to improve intestinal microbiota homeostasis, maintain gut barrier integrity, and modulate immune response (Figure 1), and exert beneficial effect to host health by preventing against pathogen invasion and risks of obesity, type 2 diabetes, inflammatory bowel disease, cancer, cardiovascular, liver, and central nervous system disorders (4).

## Effects of Probiotics on Gut Microbiota and Immune Function

At the beginning of the 1900s, Metchnikoff firstly introduced the scientific probiotic concept and hypothesised that longevity in Bulgarian peasants stemmed from their large intakes of fermented milk containing health-promoting microorganisms (now known as probiotics) (5). In 2001 and 2002, probiotics were defined as “live microorganisms that, when administered in adequate amounts, confer a health benefit on the host” by a WHO/FAO working party (6). In 2014, probiotics were stipulated as “defined contents, appropriate viable count at end of shelf life, and suitable evidence for health benefits” and further stated that all probiotics must be “safe for their intended use” by Hill et al. (6). These criteria were then reiterated by the International Scientific Association of Probiotics and Pre-biotics (ISAPP) in a position statement in 2018 (5). Up to now, the list of probiotics that can be used in food includes 35 species or subspecies and has been divided into three categories: lactic acid-producing bacteria (*Lactobacillus, Bifidobacterium*, and *Enterococcus*), yeast, and spore-forming *Bacillus* species. *Bifidobacterium* spp. and *Lactobacillus* spp. are generally isolated from healthy human colon or dairy sources (7).

### Effects of Probiotics on Gut Microbiota

It is necessary to maintain the balance of the gastrointestinal microbiome, which plays a significant role in many diseases. The gastrointestinal epithelium is the first point of host contact with microorganisms, the infection, and invasion of any pathogenic bacteria will contact the mucosa of the intestinal tract and activate immune response (8). Changes in the composition and diversity of microflora are associated with several gastrointestinal diseases (inflammatory bowel disease, colorectal cancer, or irritable bowel syndrome) and parenteral diseases, such as allergies, bronchial asthma, and cystic fibrosis (9, 10).

Probiotics have been shown to influence gut microbiota in animal studies and human clinical trials (Table 1). *Bifidobacterium* spp. are primary beneficial bacteria inhabiting in human intestines, and the alterations in the number and composition of microbiota are one of the most common features of diseases, such as Crohn's disease (24), ulcerative colitis (25), respiratory infection disease (11), and autism (26). Probiotics could modulate human intestinal bacteria, inhibiting harmful bacteria, such as *Desulfovibrio*, and stimulating beneficial bacteria, such as lactic acid bacteria. In one study, probiotic Bifico (*Bifidobacterium infantis, Lactobacillus acidophilus, Enterococcus faecalis*, and *Bacillus cereus*) pre-treatment has been found to reduce the risks of colitis-associated cancer and formation of tumour in mice, through decreasing the abundance of colitogenic bacteria, such as *Desulfovibrio, Mucispirillum*, and *Odoribacter*, and increasing the abundance of *Lactobacillus* (12). In another animal study, probiotic yeast *Saccharomyces boulardii* (*S. boulardii*) was found to significantly increase the abundance of *Bacteroidetes* and decrease the abundance of *Firmicutes, Proteobacteria*, and *Tenericutes*, which were correlated with the host metabolism alterations, and indicated the potential role of *S. boulardii* in the treatment of obesity and type 2 diabetes (13). The impacts of probiotics on gut microbiota are also investigated and proved in human clinical trials. In a randomised, double-blind, and placebo-controlled human dietary intervention study, three different doses [5 × 10^9^ CFU/day (high), 1.0 × 10^9^ CFU/day (medium), and 6.5 × 10^7^ CFU/day (low)] of *B. lactis* HN019 were found to significantly increase levels of bifidobacteria and lactobacilli in elderly compared to placebo. Importantly, this study demonstrated that the lowest dose of *B. lactis* HN019 (6.5 × 10^7^ CFU/day) was effective in microbiota modulation in the elderly, provided *B. lactis* HN019 consumption guidelines (14). The mixture of probiotic strains not only showed gut microbiota modulatory effect in healthy volunteers (15) but also in volunteers with recurrent respiratory tract infection disease (11). According to these studies, probiotics could regulate and restore gut microbiota balance through stimulating the growth/activity of beneficial bacteria and suppressing those of harmful bacteria. Although the underlying mechanism of probiotics on gut microbiota modulation remains unclear, probiotics could inhibit the growth of pathogens through the production of short-chain fatty acids (SCFA) and toxins (27) and competition of colonisation sites with pathogens (28). In addition, as more next-generation probiotic strains are identified, the strain-specific effect and effective dose of them need to be investigated.

**Table 1:** Example of studies designed to determine effects of probiotics on gut microbiota and immune function.

**Subjects**	**Substrates**	**Dose**	**Duration**	**Results**	**References**
66 healthy infants	Infant formulas (*Bifidobacterium infantis* R0033, *B. bifidum* R0071, and *Lactobacillus helveticus* R0052)	Feeding >80% of daily food	4 weeks	High levels of faecal sIgA, suggesting a positive effect of probiotics on sIgA production	(9)
120 children	*Bifidobaeterium* tetravaccine tablets (*B. infantis, L. acidophilus, Enterococcus faecalis* and *Bacillus cereus*)	3 tablets/12 h	2 months	The number of *Bifidobacterium* and *Lactobacillus* was significantly higher	(11)
35 C57BL/6 mice	Probiotic cocktail Bifico (*B. longum, L. acidophilus, Enterococcus faecalis*)	1.2 × 10^7^ CFU/d	9 weeks	Bifico decreased the abundance of genera *Desulfovibrio, Mucispirillum*, and *Odoribacter*, and a bloom of genus *Lactobacillus* was detected	(12)
30 6-week-old db/db mice	*Saccharomyces boulardii Biocodex*	120 mg/d	4 weeks	Significantly change the gut microbiota composition with an increased abundance of *Bacteroidetes* and a decreased abundance of the phyla *Firmicutes, Proteobacteria*, and *Tenericutes*	(13)
80 elderly people	*B. lactis* HN019	5 × 10^9^ CFU/d, 1.0 × 10^9^ CFU/d, and 6.5 × 10^7^ CFU/d	4 weeks	All the three doses caused a significant increase in *Bifidobacteria, lactobacilli* and *enterococci* and a decrease in *Enterobacteriaceae*	(14)
20 healthy Italian volunteers	*B. longum* BB53*6, L. rhamnosus* HN001	4 × 10^9^ CFU/d	4 weeks	A higher abundance of *Blautia producta, Blautia wexlerae* and *Haemophilus ducrey* was observed, together with a reduction of *Holdemania filiformis, Escherichia vulneris, Gemmiger formicilis* and *Streptococcus sinensis abundance*	(15)
Macrophages derived from monocytes	*L. rhamnosus* GG, *L. rhamnosus* KLSD, *L. helveticus* IMAU70129*, and L. casei* IMAU60214	10^8^ CFU/mL/d	24 h	Improve the phagocytosis and bactericidal activity such as *S. aureus, S. typhimurium*, and *E. coli* (*Staphylococcus aureus, Salmonella typhi-murium, E. coli*)	(16)
20 Balb/c mice	*L. gasseri* SBT2055 (LG2055)	1.0 × 10^9^ CFU/g	5 weeks	An increased production of IgA and numbers of IgA+ cells in Peyer's patches and lamina propria	(17)
30 BALB/c mice	*L. acidophilus, L. casei, L. reuteri, B. bifidium, and Streptococcus thermophilus*	5 × 10^8^ CFU/d	20 days	Increased numbers of CD4^+^Foxp3^+^ regulatory T cells, and decrease numbers of Th 1, Th2, and Th17 cytokines	(18)
44 healthy adults	*B. animalis*	10^9^CFU/d	21 days	Probiotic combined with xylo-oligosaccharide could reduce the expression of CD19	(19)
47 healthy women	Total bacteria in human milk	1.5 to 4.0 log^10^CFU/mL	24 h	No potential probiotics were found to antagonise pathogens, but they all agglutinate different pathogens	(20)
20 BALB/c mices	*Lactobacilli* and *Bifidobacteria*	5 × 10 ^9^ CFU/mL/d	6 days	Blocking autophagy *in vitro* reduces the IL-10 and exacerbates the secretion of IL-1β	(21)
66 adult males	*B. bifidum* and *L. plantarum*	*Bifidobacteria*: 7.5 log CFU/g/d *Lactobacilli*: 4.59 log CFU/g/d	1 week	*Lactobacillus* and *Enterococcus* significantly increased from day 1 to day 7	(22)
180 people	*Streptococcus thermophilus* MG510 and *L. plantarum* LRCC5193	*Streptococcus thermophilus* MG510: 3.0 × 10^8^ CFU/g/d and *L. plantarum* LRCC5193: 1.0 × 10^8^ CFU/g/d	4 weeks	The relative abundance of *L*. plantarum remained higher in the probiotic group than in the placebo group at 8 weeks, no increment of *Streptococcus thermophilus* was observed in the faecal microbiota	(23)

*IgA, immunoglobulin A; sIgA, secretory immunoglobulin A; T cells, Tregs; Th 1, T helper; IL-10, interleukin 10*.

### Effects of Probiotics on Immune Function

Pattern recognition receptors, such as toll-like receptors (TLR), which are expressed on immune cells, could be recognised by probiotics, after which probiotics could regulate important signalling pathways, producing nuclear factor κb (NF κb) and mitogen-activated protein kinases, and communicate with the host (29). In addition, the innate immune response could be activated, resulting in the production of pro- and anti-inflammatory cytokines or chemokines (8). The effects of probiotics on immunity are summarised in Table 1.

In an *in vitro* study, four different strains of probiotics [*Lactobacillus rhamnosus* (*L. rhamnosus*) GG, *L. rhamnosus* KLSD*, Lactobacillus helveticus* (*L. helveticus*) IMAU70129, and *Lacticaseibacillus casei* (*L. casei*) IMAU60214] could stimulate innate immunity by increasing phagocytosis of human monocyte-derived macrophages, levels of reactive oxygen species (ROS), and signalling of NF-κB pp65 and TLR2 (16). Another *in vivo* study also found similar results, the pre-treatment of *Lactobacillus johnsonii* (*L. johnsonii)* NBRC 13952 could enhance the phagocytosis of macrophage cell line RAW264.7 on various pathogens and promote the expression of interleukin-1β (IL-1β) and CD80 (30). The regulatory effects were further investigated in animal and human studies. In an animal study, mice orally administrated with *Lactobacillus gasseri* (*L. gasseri*) SBT2055 (LG2055) were found to have increased production of IgA and numbers of IgA+ cells in Peyer's patches and lamina propria, which might result from the stimulation of transforming growth factor β (TGF-β) expression and activation of TLR2 signalling pathways (17). Kwon et al. (18) applied the animal model and found probiotics mixtures *L. acidophilus, L. casei, Limosilactobacillus reuteri* (*L. reuteri*)*, Bifidobacterium bifidum* (*B. bifidium*)*, and Streptococcus thermophilus* (5 × 10^8^ cfu/day) could increase numbers of CD4 + Foxp3+ regulatory T cells (Tregs) and decrease numbers of T helper (Th) 1, Th2, and Th17 cytokines, contributing to inhibit progression and immune disorders in inflammatory bowel disease, atopic dermatitis, and rheumatoid arthritis. *Bifidobacterium breve* (*B. breve*) AH1205 and *Bifidobacterium longum* (*B. longum*) AH1206 also could promote the expression of transcription factor Foxp3 to induce Tregs in infant mice, which were associated with a protective effect against allergy (31). A human clinical trial also showed that healthy infants fed with infant formulas with *B. infantis* R0033, *Bifidobacterium bifidum* (*B. bifidum*) R0071, and *L. helveticus* R005 would have a higher level of faecal secretory IgA (sIgA) compared to control, which were associated with enhanced mucosal immunity (9). In addition, probiotic *B. infantis* 35624 has been shown to increase the proportion of Foxp3^+^ lymphocytes in peripheral blood in healthy volunteers and decrease levels of pro-inflammatory cytokines and C-reactive protein in psoriasis patients, chronic fatigue syndrome patients, or ulcerative colitis patients (32, 33). Childs and Röytiö (19) conducted a double-blind, placebo-controlled, randomised, factorial cross-over study to investigate the impact of probiotic *Bifidobacterium animalis* (*B. animalis*) on immune response and found that probiotics combined with xylo-oligosaccharide could reduce the expression of CD19 on B cells. These findings suggested the crucial role of probiotics in immune function regulation, not only in healthy but also in disease patients through activating important immune signalling pathways and modulating the activity of immune cells. Importantly, the role of probiotics as alternative supplementation in disease treatment and the underlying mechanisms need to be further investigated.

## Effects of Pre-biotics on Gut Microbiota and Immune Function

The concept of pre-biotics was introduced by Gibson and Roberfroid when they observed certain non-digestible oligosaccharides were selectively fermented by bifidobacteria (34). The concept of pre-biotics was firstly proposed as a selectively fermented ingredient that results in specific changes, in the composition and/or activity of the gastrointestinal microbiota, thus conferring benefit(s) upon host health (35). Subsequently, with the improved knowledge of gut microbiota composition and underlying mechanisms of pre-biotics (36), the definition of pre-biotics was updated in 2017 as “a substrate that is selectively utilised by host microorganisms conferring a health benefit” by an ISAPP consensus panel led by Gibson (5). Pre-biotics, such as fructooligosaccharides, galactooligosaccharides (GOS), and lactulose, have been found to significantly modulate the balance of the large intestine microorganism community by increasing the number of bifidobacteria and lactic acid bacteria (37). Other nutrients, such as pectin, cellulose, and xylan, are also beneficial to the balance of intestinal microorganisms (38). Pre-biotics play an important role in the prevention of diarrhoea (39), constipation (40), metabolic disease (41), and cancer (42) and confer positive effects on lipid metabolism (43), mineral adsorption (44), and immune regulation (45).

### Effect of Pre-biotics on Gut Microbiota

Pre-biotics could regulate the balance of intestinal microorganisms and improve colon health (46–49) (Table 2). Inulin-type fructans (ITFs) have shown bifidogenic effects in *in vitro* and human studies (46). Inulin was found to increase bifidobacteria and lactobacilli in faecal samples from the elderly population (67, 68) and healthy adults (69) *in vitro*. In a human study, 30 obese women were recruited and randomly divided into 2 groups, one group daily received 16g ITF (*n* = 15), the other group maltodextrin (*n* = 15) for 3 months. Qualitative and quantitative analyses of faecal microbiota found that ITF intervention increased levels of *Bifidobacterium* and *Faecalibacterium prausnitzii* and decreased levels of *Bacteroides intestinalis and Bacteroides vulgate* compared to maltodextrin (50). In healthy adults, inulin could also modulate gut microbiota composition, with a significant increase in the numbers of bifidobacteria (70). Although inulin has shown modulatory impact on gut microbiota, the impact is related to the degree of polymerization (DP) of inulin. In a mice model study, high-fat-diet feeding obesity mice received inulin with DP ≤ 9 and DP ≥ 23, respectively, for 8 weeks. The results showed the inulin with longer DP could decrease the abundance of *Firmicutes* and increase the abundance of *Bacteroidetes* more significantly and perform a more beneficial impact on liver injury (71). In addition, the impact of GOS on gut microbiota was also well-studied. In a mice study, the effect of GOS in prevention and alleviation against *Escherichia coli* (*E. coli*) O157 invasion and colonisation was studied. The results showed that GOS could stimulate the growth and activities of beneficial bacteria, such as *Akkermansia, Ruminococcaceae*, and *Bacteroides*, and promote the production of SCFA (51). GOS could also increase the abundance of *Lactobacillus* and *Lactococcus* in rats with constipation compared to placebo, hence suppression of constipation and exerting a beneficial impact on colon health (72). In addition, GOS has also been shown to modulate gut microbiota with bifidogenic effect in volunteers with gastrointestinal symptoms, such as bloating, flatulence (73), autism (74), and those aged over 60 (67, 68). Other pre-biotics also showed a modulatory impact on gastrointestinal microorganisms. Pre-biotic Mushroom, *Bulgaria inquinans* (BI) was assessed in male C57BL/6 mice to investigate its impact on gut microbiota. Mice received BI (1 or 2%) for 4 weeks, and the results indicated the decreased diversity of gastrointestinal bacteria, increased abundance of *Faecalibaculum* and *Parabacteroides*, and decreased abundance of *Allobaculum* and *Rikenella* (52). A study showed that moderate intake of red wine polyphenols could regulate gut microbiota composition in patients with metabolic syndrome, with significantly increased the number of *Bifidobacterium, Lactobacillus*, and butyrate-producing bacteria (*Faecalibacterium prausnitzii* and *Roseburia*) in faeces (41). In a 100 healthy adult study, volunteers consumed placebo, 2′-O-fucosyllactose (2′FL), lacto-N-neotetraose (LNnT), or 2′FL+LNnT (2:1 mass ratio; mix) at 5, 10, or 20 g daily. The results showed that 2′FL and/or LNnT could significantly increase the relative abundance of *Actinomycetes* and *Bifidobacterium* and decrease those of *Firmicutes* and *Proteobacteria*, hence maintaining gut microbiota balance (53). Although the gastrointestinal microbiota can be modulated by pre-biotics, individual responses can vary. For example, a study aimed to investigate the impact of GOS (0.0, 2.5, 5.0, and 10.0 g GOS) on faecal microbiota of healthy human subjects found that GOS mainly increased the abundance of organisms within the *Actinobacteria*. However, only 50% of these changes can be detected by individuals. In addition, the response to the GOS varies from individual to individual (75). According to these findings, pre-biotics have been shown to modulate microbiota composition, and these effects could result in alleviating symptoms observed in patients. In addition, different pre-biotics are specific to different bacteria and their effect doses are also different. Therefore, it is important to make scientific results and conclusions reliable and develop proper instructions for consumers.

**Table 2:** Example of studies designed to determine effects of pre-biotics on gut microbiota and immune function.

**Subjects**	**Substrates**	**Dose**	**Duration**	**Results**	**References**
10 patients with metabolic syndrome and 10 healthy subjects	Polyphenols	272 mL/d	1 month	Significantly increased the number of *Bifidobacterium, Lactobacillus*, and butyrate-producing bacteria (*Faecalibacterium prausnitzii* and *Roseburia*) in faeces	(41)
90 men and 50 women	Ructooligosaccharides, xylooligosaccharides, polydextrose, and resistant dextrin	30 g/d	7 days	Increased serum IgG, IgM and transferrin, increased the abundance of *Bifidobacterium* and *Enterococcus*, decreased the abundance of *Bacteroides*	(42)
30 obese women	ITF	16 g/d	3 months	Increased levels of *Bifidobacterium* and *Faecalibacterium prausnitzii*, and decreased levels of *Bacteroides intestinalis and Bacteroides vulgate*	(50)
30 BALB/c mice	GOS	0.2 g GOS/100 g body weight	3 weeks	GOS could stimulate the growth and activities of beneficial bacteria, such as *Akkermansia, Ruminococcaceae*, and *Bacteroides*, and promote the production of SCFA	(51)
18 C57BL/6 mice	BI	1 or 2%/d	4 weeks	Decreased diversity of gastrointestinal bacteria, increased abundance of *Faecalibaculum* and *Parabacteroides*, and decreased abundance of *Allobaculum*, and *Rikenella*	(52)
100 healthy adult study	2′FL LNnT	5, 10, 20 g/d	2 weeks	2′FL and/or LNnT could significantly increase relative abundance of *Actinomycetes, Bifidobacterium*, and decrease those of Firmicutes and Proteobacteria	(53)
113 pre-term infants [gestational age <32 weeks, birth weight (BW) <1,500 g]	11% inulin-enriched pasta	1.5 g/kg/d	30 days	Could improve barrier function of the gut, with significantly higher levels of glucagon-like peptide-2 and lower levels of zonulin in serum	(54)
114 Obese adults	GOS	5 g/d	4 weeks	Decreased sucralose excretion compared to placebo	(55)
47 Obese volunteers	AX	7.5 and 15 g/d	6 weeks	Did not significantly improve gastrointestinal permeability	(56)
34 crossbred Yorkshire-Landrace pigs	Inulin	10% (w/w) long-chain purified chicory inulin-enriched diet	2 weeks	Increased expressions of Th2-related immune genes, such as IL-13 and IL-5, and declined expressions of Th2-related immune genes, such as IL-1α and IL-8	(57)
16 SPF HLA-B27 transgenic rats	Inulin (5 g/kg with water)	30 ml/d	7 weeks	Could reduce the levels of IL-1β and IFN-γ, suggesting the immunomodulatory impact of inulin	(58)
52 women with type 2 diabetes	Inulin	10 g/d	8 weeks	Significantly declined levels of IL-6, and lipopolysaccharide	(59)
12 non-diabetic overweight adults	Inulin	20 g/d	44 days	Inulin had no effect on the systemic inflammatory indexes studied	(60)
40 healthy volunteers (18–29 years)	DP10-60, DP2-25 inulin	8g/d	35 days	DP10-60 inulin could more significantly induced the production of Th1-related cytokines and the activation of TLR2 compared to DP2-25 inulin. In addition, DP10-60 inulin could increase the numbers of B cells and Th1-cells, and the titre of anti-HBsAg, whereas DP2-25 could not	(61)
40 elderly	B-GOS	5.5 g/d	10 weeks	Could increase levels of IL-8, IL-10, and C-reactive protein, decrease levels of IL-1β, and stimulate activities of NK cell	(62)
60 pre-hypertensive males	OLE (oleuropein; hydroxytyrosol)	136, 6 mg/d	6 weeks	Reduced plasma levels of IL-8 in pre-hypertensive volunteers	(63)
30 women (BMI > 30 kg/m^2^, 18–65 years)	Inulin + oligofructose	16 g/d	12 weeks	Increased *B. longum, B. pseudocatenulatum*, and *B. adolescentis*	(64)
49 healthy adults	Long-chain inulin and oligofructose	50:50 mixture 8 g/d	8 weeks	NK cell activity, immunocyte phenotype bactericidal activity and T cell activity was increased	(65)
57 healthy adults	Short chain galactooligosaccharides/long chain fructooligosaccharides/pectin hydrolysate-derived acidic oligosaccharides (scGOS/lcFOS/pAOS)	15 or 30 g/d	12 weeks	Increased *Bifidobacteria*, decreased *Clostridium coccoides/Eubacterium* rectale cluster. Increased NK cell activity, reduced activation of CD14, CD25	(66)

*ITF, inulin-type fructan; SCFAs, short-chain fatty acids; GOS, galactooligosaccharide; B-GOS, galacto-oligosaccharide mixture; 2′FL, 2′-O-fucosyllactose; LNnT, lacto-N-neotetraose; BI Mushroom Bulgaria inquinans; IFN-γ, interferon-gamma; AX, arabinoxylans; DP, degree of polymerization*.

### Effects of Pre-biotics on Immune Function

#### Protective Effect of Pre-biotics on Intestinal Epithelial Barrier

The protective effects of pre-biotics on intestinal epithelium have been confirmed, and host immunity can be enhanced by improving the integrity of intestinal epithelium (76). In a human study, 11% inulin-enriched pasta could improve the barrier function of the gut, with significantly higher levels of glucagon-like peptide-2 and lower levels of zonulin in serum, hence protecting mucosal barrier integrity and preventing gastrointestinal diseases (54). GOS could also help maintain gut barrier function and improve colon permeability. Obese adults who received 5 g/d GOS for 4 weeks showed a decreased sucralose excretion compared to placebo, suggesting improved barrier function induced by GOS (55). Similar results of GOS were also found in preterm infants (77). Although inulin and GOS could help maintain gut barrier integrity, the impacts of arabinoxylans (AX) remain controversial. In one human study, two different doses of AX (7.5 and 15 g/d) did not significantly improve gastrointestinal permeability in obese volunteers (56). Therefore, the impact of pre-biotics on the integrity and function of the intestinal epithelial barrier needs to be further assessed.

#### Effects of Pre-biotics on Immune Response

Pre-biotics may also contribute to the regulation of immune response through inhibiting expressions of pro-inflammatory cytokines, stimulating those of anti-inflammatory cytokines, and promoting activities of immune cells, such as macrophages, NK cells, T cells, and B cells (Table 2). In a porcine model study, inulin was found to induce an anti-inflammatory immune response against pathogen infection, with increased expressions of Th2-related immune genes, such as IL-13 and IL-5, and declined expressions of Th2-related immune genes, such as IFNG, IL-1α, and IL-8, which were closely related with microbiota composition (57). In another animal study, 16 SPF HLA-B27 transgenic rats were divided into two groups, the inulin group fed with the combination of chicory-derived long-chain ITFs and short-chain inulin fraction oligofructose, and the control group fed with placebo for 7 weeks. The caecum and colon tissue were collected and analysed for cytokine production. The results showed that pre-biotic intervention could reduce the levels of IL-1β and interferon-gamma (IFN-γ), suggesting the immunomodulatory impact of inulin (58). In addition, inulin could also help to regulate immune markers in patients with type 2 diabetes, with significantly declined levels of tumour necrosis factor-α (TNF-α), IL-6, and lipopolysaccharide (59). However, inulin supplementation could not regulate systemic inflammatory markers in a human study, in which twelve non-diabetic overweight adults daily received 20 g inulin for 44 days (60), the results showed that inulin had no impact on IL-8. Notably, the immunomodulatory impact of inulin depends on the DP or chain length. A double-blind, placebo-controlled human study found that DP10-60 inulin could more significantly induce the production of Th1-related cytokines and the activation of TLR2 compared to DP2-25 inulin. In addition, DP10-60 inulin could increase the numbers of B cells, Th1-cells, and the titre of anti-HBsAg, whereas DP2-25 could not (61). The impact of GOS on the immune response is also investigated. A study, which assessed the influence of Bimuno-GOS (B-GOS) on immune function of 40 elderly, found 5.5 g daily intake of B-GOS for 10 weeks could increase levels of IL-8, IL-10, and C-reactive protein, decrease levels of IL-1β, and stimulate activities of NK cells, suggesting the beneficial effects induced by B-GOS supplementation on ageing population (62). The results were in agreement with previous studies on overweight adults (78), elderly volunteers (79), and *in vitro* studies (67, 68). Not only inulin and GOS but other pre-biotics also showed immunomodulatory activity. Pre-biotic Mushroom BI was assessed in male C57BL/6 mice to investigate its impact on the host immune response. The results showed that 1 and 2% BI intervention could stimulate the proliferation of T cells from the spleen, and 2% BI could even increase IL-2 production in splenocytes, consequently influencing the peripheral and mucosal immune systems (52). In a randomised, controlled study, polyphenol supplementation reduced plasma levels of IL-8 in pre-hypertensive volunteers (63). In summary, pre-biotics could enhance host immunity in health and disease patients through protecting intestinal barrier function, activating immune cells, and regulating immune signalling pathways.

## Effects of Post-biotics on Gut Microbiota and Immune Function

Post-biotics include cell wall components, such as protein molecules and lipopolysaccharides, extracellular polysaccharides, and microbial metabolites of carbohydrate fermentation or protein degradation, such as SCFA and branched chain fatty acids (80). Several studies have found that post-biotics can exert positive biological functions to the host (4, 81).

### Effect of Post-biotic on Gut Microbiota

Post-biotic has great potential to maintain homeostasis of intestinal microbiota and improve intestinal health, through inhibiting the growth and activities of harmful bacteria and stimulating those of beneficial bacteria (Table 3). The impact of metabolic products of probiotic fermentation is well-studied. Cell-free spent media (CFSM) of six probiotics (*L. acidophilus* EMCC 1324, *L. helveticus* EMCC 1654, *L. plantarum ss. plantarum* EMCC 1027, *L. rhamnosus* EMCC 1105, *B. longum* EMCC 1547, and *B. bifidum* EMCC 1334) have been shown to exert strong antibacterial activity to *Escherichia coli* isolates, with inhibition zones of 11.77–23.10 mm. Notably, CFSM of *L. plantarum* had the strongest antibacterial activity compared to the other five, with a 64.57% reduction in biofilms of *E. coli* (88). In another *in vitro* study, CFSM and biofilm of probiotics *L. rhamnosus* and *L. casei* also performed an antifungal activity to *Candida albicans* (89). Similar findings were also observed in cell-free supernatant (CFS) from *L. kunkeei* against *Candida albicans* (90). CFS from *L. paracasei* CNCM I-4034, *B. breve* CNCM I-4035, and *L. rhamnosus* CNCM I-4036 isolated from a faecal sample of breast-feeding infants could suppress the growth of *E. coli, Salmonella*, and *Shigella* over 50%, and *L. paracasei* CNCM I-4034 CFS showed the strongest antimicrobial activity (81%) *in vivo*. However, when CFS was neutralised, decreased antimicrobial activities were observed, indicating the important role of organic acid and antimicrobial substances produced by probiotic fermentation (91). CFS from probiotic *L. rhamnosus* GG also exhibited a strong antibacterial activity against *E. coli* K1, the adhesion, invasion, and translocation of which have been shown to be blocked by CFS *in vitro*, through stimulating the production of mucin and protecting intestinal barrier function. This was demonstrated in the neonatal rat model, CFS could protect neonatal rats from *E. coli* K1 infection, through increasing expression levels of Ki67, MUC2, ZO-1, IgA, and mucin and decreasing intestinal barrier permeability (92). Besides, in a broiler model study, the impact of the mixture of CFS from *L. plantarum* RG14 and inulin on colon health and immune function was investigated. Increased numbers of total caecal microbiota and bifidobacteria, and decreased number of *Enterobacteria* and *E. coli*, were observed in birds fed with CFS and inulin compared to birds fed with placebo. In addition, CFS and inulin could stimulate the production of acetic acid in birds, besides, the immune markers were regulated by CFS and inulin, resulting in decreased levels of IFN and TNF-α and increased levels of IL-6. The results illustrated the potential role of post-biotic CFS from *L. plantarum* RG14 in growth promoters in the poultry industry (82). CFS from *L. plantarum* RG14 could also improve the nutrients digestion and absorption in newly-weaned lambs, suggested by increasing the number of fibre degrading bacteria, such as *Fibrobacter succinogenes* and *Ruminococcus flavefaciens*, and expressions of IGF-1 and MCT-1 (93). These findings demonstrated the antimicrobial and microbiota regulatory activity of post-biotics, however, the bioactive compounds were not identified and separated.

**Table 3:** Example of studies designed to determine effects of post-biotic on gut microbiota and immune function.

**Subjects**	**Substrates**	**Dose**	**Duration**	**Results**	**References**
261 COBB 500 chicks	Inulin	1%/d	6 weeks	Increased number of total caecal microbiota and *Bifdidobacteria*, and decreased number of *Enterobacteria* and *E. coli*. In addition, CFS and inulin could stimulate the production of acetic acid in birds	(82)
12 male lambs	RG14, RG11, and TL1 from Lactobacillus plantarum	0.9%/d	60 days	Regulate barrier integrity and function in lams through increased levels of tight junction protein, occludin, claudin-1	(83)
40 C57BL mice	Post-biotic HM0539 from LGG	10 μg/d	7 days	COX-2, and iNOS, subsequently suppressing production of PGE2 and NO	(84)
300 male broilers	Post-biotics from *Saccharomyces cerevisiae* fermentation	10%/d	35 days	Decrease levels of IL-6 and IL-1ß compared to control, and increase levels of tight junction protein, occludin, and claudin-1.	(85)
Murine macrophage cell line, J774A.1 cell	EPS was isolated from *B. longum* BCRC 14634	5 μg/mL	24–48h	The proliferation of J77A.1 macrophage and their secretion of the anti-inflammatory cytokine IL-10 was elevated	(86)
*Staphylococcus aureus* and *Enterobacter aerogenes* of 220.25 ± 3.3 and 170.2 ± 4.6 AU/mL	CFNS of associated *Staphylococcus succinus* strain (AAS2)	Exopolysaccharide (41.3 ± 0.6 mg/L/d) and lipase production (8.3 ± 0.3 mm/d)	24h	Moderate level of exopolysaccharide and lipase production can reduce the viability of *Staphylococcus aureus* and *Escherichia aerogenes*	(66)
Ctreg isolated from 90 GF mices	SCFA production of species belonging to Clostridium cluster XI, XIV, XVII	Propionate (14–62 vs. 0.05–1.1 μmol/10^5^ CFU) and acetate (118–220 vs. 0.1–2 μmol/10^5^ CFU)	3 weeks	Treatment significantly increased foxp3 and IL-10 expression, this suggests that SCFA specificity induces the Treg of foxp3 and IL-10 production	(87)

*CFNS, cell-free neutralised supernatant; IL-6, interleukin; COX-2, Inhibition effects on cyclooxygenase 2; iNOS, inducible nitric oxide synthase; SCFAs, short-chain fatty acids; GOS, galactooligosaccharide; PGE2, prostaglandin E2; NO, nitric oxide; IL-10, interleukin-10*.

To further explore the underlying mechanism, a few research groups focused on the exopolysaccharides (EPS), and proteins, etc. To investigate the impact of EPS on gut microbiota, an *in vitro* study applied a batch culture model and compared eleven EPS isolated from different human bifidobacteria with glucose and inulin. The results illustrated that EPS and inulin could stimulate the growth of bifidobacteria and the production of SCFA, resulting in a decreased ratio of acetic acid/propionic acid, which was contrary to glucose fermentation. In addition, the strain-specific impact of EPS was indicated, as EPS from *B. pseudocatenulatum* contributed to the growth of *Desulfovibrio* and *Faecalibacterium prausnitzii*, whereas EPS from *B. longum* contributed to the growth of *Anaerostipes, Prevotella*, and/or *Oscillospira* (94). The results showed EPS from bacteria could be utilised by gastrointestinal microorganisms, hence affecting colon health. The post-biotic could also inhibit the adhesion of pathogens to epithelial cells. A novel soluble protein HM0539 from *L. rhamnosus* could inhibit the adhesion and invasion of *E. coli* O157: H7 to HT-29 cell dose-dependently (95). Kaikiri et al. (96) also found that a novel gut microbiota metabolite 10-hydroxy-cis-12-octadecenoic acid (HYA) could alter the faecal microbiota community of NC/Nga mice, although the PCR-denaturing gradient gel electrophoresis analysis did not show detailed bacteria species change induced by HYA. The impact of post-biotics on bacteria is mainly demonstrated *in vitro* and *in vivo*, hence further evidence from human clinical trials is required, and post-biotics could be used as alternative antimicrobial compounds and supplementation strategies for disease alleviation or treatment.

### Effects of Post-biotics on Immune Function

#### Protective Effect of Post-biotics on Intestinal Epithelial Barrier

Post-biotics could modulate host immunity by improving gastrointestinal barrier function and inhibiting pathogen translocation. In one study, CFS from *L. plantarum* fermentation could regulate barrier integrity and function in lambs through increasing levels of tight junction protein, occludin, claudin-1, and CLDN-4 (83). The bioactive compound HM0539 has also been shown to stimulate mucin expression and decline expression levels of MUC2 and zonula occludens-1 (ZO-1) in the neonatal rat model, indicating its intestinal barrier protecting role (97). As shown in the above studies, it is mainly the increased tight junction protein induced by post-biotics that illustrated its positive role in the improvement of intestinal epithelial barrier function. Hence, further immunological, biochemical, and pathological section evaluation from clinical trials is warranted to support this view.

#### Effects of Post-biotics on Immune Response

Post-biotics may affect the innate and adaptive immune system through the interaction of many cell types along the mucosa, such as B cells, T cells, monocytes, macrophages, NK cells, and dendritic cells (DCs) (98). Cell wall components, including peptidoglycan (99), have been shown could bind to receptors on the surface of monocytes and macrophages, consequently stimulating immune cells to produce cytokines indirectly (100, 101). Tryptophan metabolites can inhibit inflammation by acting on T cell aromatics receptors and stimulating DCs to induce Treg activation through retinoic acid (102).

In addition, post-biotics could influence immune response by affecting the immune signalling pathway through modulating inflammatory cytokines. Butyrate, an important bacteria fermentation product, was shown to facilitate monocyte polarised to M2 macrophage and suppress the pro-inflammatory immune response in mice, with increased expression of Arg1 and activation of H3K9/STAT6 signalling pathway (101). Lipoteichoic acid produced by *L. plantarum* was shown to inhibit inflammation response induced by the viral pathogen in porcine intestinal epithelial cells. The results showed lipoteichoic acid could reduce levels of IL-8 and suppress ERK phosphorylation, p38 kinase, and NF-κB activation in a dose-dependent manner (103). The post-biotic HM0539 not only exhibited an inhibition effect against the pathogens but also an immunomodulatory effect. In *in vitro* cell culture and dextran sulphate sodium (DSS)-induced murine colitis model, HM0539 showed an inhibition effect on cyclooxygenase 2 (COX-2), and inducible nitric oxide synthase (iNOS), subsequently suppressing the production of prostaglandin E2 (PGE2) and nitric oxide (NO), TLR4-MyD88, and NF-κB signalling pathways, and hence might serve as a candidate strategy in inflammatory bowel disease treatment (84). Metabolic products from *Pediococcus acidilactici, L. reuteri, E. faecium*, and *L. acidophilus* fermentation were assessed in broiler chicks challenged with *Clostridium perfringens* infection. The immunomodulatory activity of these metabolic products was illustrated by the downregulating pro-inflammatory response, especially the Kyoto Encyclopaedia of Genes and Genomes (KEGG) signalling pathways (104). An animal experiment investigated the influence of post-biotics from *Saccharomyces cerevisiae* fermentation product in male broilers and found they could decrease levels of IL-6, NF-κB, and IL-1ß compared to control, and increase levels of tight junction protein, occludin, and claudin-1, indicating their anti-inflammatory activities and protective role in barrier function (85). An *in vivo* study also showed that EPS from *B. animalis* subsp. *Lactis* could modulate the immune response, inducing the downregulation of pro-inflammatory cytokines (105). In *ex vivo* cultures of mucosa from diarrhoea patients, CFS of *L. casei* DG could downregulate pro-inflammatory immune response, upregulate anti-inflammatory immune response, *via* reducing expression levels of IL-1α, IL-6, IL-8, and TLR-4, and increasing those of IL-10 (106).

The impacts of the cell wall on immune response were also compared by a few studies. The cell wall components of probiotic Ganeden *Bacillus coagulans* 30 (GBC30) were shown to stimulate the maturation of monocyte in human peripheral blood mononuclear cell (PBMC) culture. The shift of monocyte toward macrophage and DCs was observed with GBC30 cell wall components, with higher CD80 and CD86 expressions (107). Furthermore, an *in vivo* study indicated the immunomodulatory activity of GBC30 cell wall components through suppressing the production of ROS, increasing phagocytosis and migration of PBMC, enhancing the proportion of NK cells, stimulating the production of anti-inflammatory cytokine production, such as IL-4, and IL-10, and decreasing pro-inflammatory cytokine production, such as TNF-α and IFN-γ, consequently balancing Th1/Th2 immune response (86). The immunomodulatory activity of cell wall components and CFS from seventeen lactic acid bacteria strains were assessed in another *in vivo* study. The results showed that IL-10 could be stimulated by CFS of all strains in peripheral blood mononuclear cells (PMBC) culture. In addition, cell wall components and CFS could both modulate the anti-inflammatory immune response, whereas, cell wall components modulate the pro-inflammatory immune response and stimulate the activity of Tregs more strongly (108).

Autophagy plays a crucial role in the innate and adaptive immune response, regulating cell homeostasis, and modulating the renewal of cellular proteins and organelles. It has recently been found an imbalance of T cells could be caused by the deficiency in autophagy, resulting in intestinal inflammation (109). The whole peptidoglycan of *B. bifidum* showed antitumour impact in BALB/c male nude mice via increasing gene expression of bax and decreasing that of bcl-2, subsequently inducing cell apoptosis (110). Peptidoglycan expressed on *L. fermentum* BGHV110 (HV110) was isolated, and its impact on autophagy was investigated in acetaminophen (APAP)-induced hepatotoxicity in HepG2 cells. The results illustrated that activation of PINK1-dependent autophagy could be stimulated by HV110 supplementation, which increased LC3 protein conversion and p62/SQSTM1 protein degradation (111).

These findings suggested that the immunomodulatory activity of post-biotic mainly depended on their ability to differentially regulate the production of anti-inflammatory and pro-inflammatory cytokines and the balance of Th1 and Th2. They can effectively regulate the gene expression of immune cells and the interference of transcription factors, thus driving the differentiation of the immune system. In addition, post-biotics may exert a significant effect on autophagy, hence influencing host immune response.

## Conclusion

This review summarised the positive impacts of probiotics, pre-biotics, and post-biotics on gastrointestinal health and immune function. Several *in vitro, in vivo*, and clinical studies have confirmed that they play a significant role in maintaining intestinal microorganism equilibrium and regulating immune response, consequently conferring benefits to host health. Although the underlying mechanism remains to be further investigated, supplementations of probiotics, pre-biotics, and post-biotics could lower colonic pH, produce antibacterial molecules, stimulate the growth and activities of beneficial bacteria, and suppress the growth of pathogens. In addition, they also play an important role in the modulation of host immunity, via improving gut barrier function, preventing pathogen translocation, altering pattern recognition receptor expression, modulating crucial signalling pathways, stimulating immune cell activities, and balancing Th1/Th2 immune response. Notably, the efficacies of probiotics, pre-, and post-biotics are typically dose-dependent, especially exhibiting species-specific for probiotic strains and structure-activity relationships for pre- and post-biotics. In addition, the profile of host microorganisms and immunity may differ based on the age, gender, exercise, health conditions, geography, etc, of the host, hence, it is necessary to consider these factors and assess increasingly quantitative and qualitative impacts of pre-biotics, probiotics, and post-biotics on gut microbiota and immune system. Besides, the underlying mechanisms should be fully illustrated via combining *in vitro, in vivo*, and clinical studies and biochemical evaluations.

## Author Contributions

All authors listed have made a substantial, direct, and intellectual contribution to the work and approved it for publication.

## Funding

This work was supported by the Science and Technology Innovation Project of Colleges and Universities of Shanxi Province (2019L0074).

## Conflict of Interest

The authors declare that the research was conducted in the absence of any commercial or financial relationships that could be construed as a potential conflict of interest.

## Publisher's Note

All claims expressed in this article are solely those of the authors and do not necessarily represent those of their affiliated organizations, or those of the publisher, the editors and the reviewers. Any product that may be evaluated in this article, or claim that may be made by its manufacturer, is not guaranteed or endorsed by the publisher.
